# Colonies of *Acropora formosa* with greater survival potential have reduced calcification rates

**DOI:** 10.1371/journal.pone.0269526

**Published:** 2022-06-09

**Authors:** Vanessa Clark, Matheus A. Mello-Athayde, Sophie Dove

**Affiliations:** 1 School of Biological Sciences, The University of Queensland, St. Lucia, Queensland, Australia; 2 ARC Centre of Excellence for Coral Reef Studies, The University of Queensland, St. Lucia, Queensland, Australia; Living Oceans Foundation, TAIWAN

## Abstract

Coral reefs are facing increasingly devasting impacts from ocean warming and acidification due to anthropogenic climate change. In addition to reducing greenhouse gas emissions, potential solutions have focused either on reducing light stress during heating, or on the potential for identifying or engineering “super corals”. A large subset of these studies, however, have tended to focus primarily on the bleaching response of corals, and assume erroneously that corals that bleach earlier in a thermal event die first. Here, we explore how survival, observable bleaching, coral skeletal growth (as branch extension and densification), and coral tissue growth (protein and lipid concentrations) varies for conspecifics collected from distinctive reef zones at Heron Island on the Southern Great Barrier Reef. A reciprocal transplantation experiment was undertaken using the dominant reef building coral (*Acropora formosa)* between the highly variable reef flat and the less variable reef slope environments. Coral colonies originating from the reef flat had higher rates of survival and amassed greater protein densities but calcified at reduced rates compared to conspecifics originating from the reef slope. The energetics of both populations however potentially benefited from greater light intensity present in the shallows. Reef flat origin corals moved to the lower light intensity of the reef slope reduced protein density and calcification rates. For *A*. *formosa*, genetic differences, or long-term entrainment to a highly variable environment, appeared to promote coral survival at the expense of calcification. The response decouples coral survival from carbonate coral reef resilience, a response that was further exacerbated by reductions in irradiance. As we begin to discuss interventions necessitated by the CO_2_ that has already been released into the atmosphere, we need to prioritise our focus on the properties that maintain valuable carbonate ecosystems. Rapid and dense calcification by corals such as branching *Acropora* is essential to the ability of carbonate coral reefs to rebound following disturbance events and maintain 3D structure but may be the first property that is sacrificed to enable coral genet survival under stress.

## Introduction

Primary (skeletal extension) and secondary (skeletal densification) calcification by reef-building corals [1,2] is critical to reef construction [3], and the ecosystem function and services provided by coral reef ecosystems [4]. However, corals (even thriving hard coral communities) are not always capable of calcifying at rates that are necessary to maintain coral reefs in a net positive carbonate balance [5]. Scleractinian corals have arguably survived past climatic events by dispensing altogether with their skeletons [6], and the coral taxa that survive present marine heatwaves appear to be those that extend at lesser rates than corals with lower survival [7]. Calcification is energetically expensive [8] and under ocean acidification potentially toxic (via acidosis and proton build up in calicoblastic epithellium) [9,10]. In some environments, it may therefore be necessary to either cease calcification or redirect energy investment away from calcification in order to facilitate coral survival [11]. Here, we evaluate drivers such as long-term pre-conditioning to thermal stress a putative epigenetic driver [12], and exposure to reduced light levels over summer and their influence on primary and secondary calcification for the branching coral, *Acropora formosa* (Dana, 1846). *Acropora formosa* is abundant in diverse zones of a coral reef where it contributes to the 3-dimensional structure and carbonate accumulation within coral reef ecosystems [13]. *A*. *formosa* is one of the most dominant branching corals found on Heron Island Reef in the Southern Great Barrier Reef (GBR) of Australia and is present on the reef slope and in lagoons typically [14]. A reef whose inhabitants are exposed to significant seasonal abiotic variation [15] and daily abiotic variation for inhabitants located on reef-flat habitats [16,17]. Underwater marine heat waves are increasing globally and are impacting multiple ecosystems including the Great Barrier Reef [18–20]. Initially, coral bleaching observed over a large area drew attention to the negative impacts ocean warming was having on coral hosts and their endosymbiotic dinoflagellates [21]. Now, more prolonged and intense thermal marine heatwaves are associated with mass coral mortality with observations suggesting that delaying the onset of bleaching in a thermal event provides no safeguard against coral mortality [7,19,22]. High rates of coral mortality along with an expected increase in the frequency of high intensity marine heatwaves suggest that future conditions will negatively impact biodiversity and services provisioned by coral communities [23]. A negative impact on coral communities is expected even under the adoption of CO_2_ mitigation pathways, such as that agreed to in Paris at the United Nations Climate Change Conference in 2015. Increases in atmospheric pCO_2_, principally due to the burning of fossil fuels, have increased the global temperature by 1°C, and equilibration of atmospheric pCO_2_ with the oceans has increased ocean acidification by 0.1 pH units, since pre-industrial times [24]. In addition to the effects of warming, more significant levels of acidification, tend to reduce the bulk density of coral skeletons, or eliminate skeleton in favour of giant fleshy standalone polyps [1,25]. Ultimately this will lead to negative outcomes for entire coral reef ecosystems which require positive calcification to maintain carbonate balances and structural complexity.

The degree to which these ecosystems will be impacted under reduced CO_2_ emission scenarios such as RCP2.6 [26] is nonetheless debated. Some argue that pre-conditioning to thermal stress can accelerate coral survival/adaptation either naturally [27] or via assisted evolution [28,29]. Preconditioning has been associated with exposure to highly variable temperature regimes that appear to confer increased tolerance to subsequent thermal stress albeit over days rather than weeks [30,31]. Some refer to this preconditioning as acclimatization [27], others as epigenetic entrainment that may be inherited by offspring providing “super corals” for the future restocking of reefs [28,32,33]. In the literature, inheritable epigenetic changes to gene expression in response to thermal stress tend to be expressed irrespectively of the presence of the stress in future habitats [12]. In this sense, epigenetic entrainment limits future transcriptional plasticity (i.e. inhibits reversion to previous state) and has been investigated in the literature through reciprocal transplantation experiments [34–36].

In literature regarding “super corals”, bleaching susceptibility (bleaching earlier rather than later as thermal stress accumulates) is often erroneously assumed to be interchangeable with mortality [36–39]. Furthermore, the potential “adaptive” costs of increased thermal tolerance on carbonate deposition are rarely considered [40,41]. Here, it is generally assumed that corals consistently retain a plasticity to accelerate rates of calcification when conditions become more favourable [3]. A lack of space to vertically accrete into is often viewed as the limiting factor when it comes to the extension rate of corals on shallow reef flats, rather than any epigenetically imposed constraints [41,42].

Other managerial or adaptive solutions to protecting reefs of the future involve the application of shade to reefs exposed to thermal stress [43]. Given that stress events often have significant longevity (e.g. 2016 Northern GBR ~3 months), the consequences of applying shading to prevent bleaching have not been fully rationalised. Shading can lead to light acclimation by the holobiont negating the benefit of reducing light exposure on symbiont reactive oxygen species (ROS) production [44]. Symbiont ROS production is linked to a bleaching mechanism in corals [45,46]. Alternatively, an absence of light acclimation in response to a reduction in light intensity would coincide with lost photosynthetic energy acquisition. A cost that may not be that dissimilar to a reduction in symbionts [47], but that might be more harmful to the host if the symbiont switch to facultative heterotrophy [48,49]. The present study was conducted at two different reef locations (habitats) (a) the shallow reef flat (1–3 m) and (b) the deeper reef slope (7–8 m) of Heron Island, in the southern GBR. Colonies from the reef flat sites are thought to experience a large diel range and variability in sea water chemistry (e.g. pCO_2_), temperature and light intensity, which is associated with the large tidal range (3m) and the resulting ponding of reefs at low tide [17,50,51]. Periods of excessively high (in summer) or low (in winter) temperatures associated with ponding can inhibit the efficiency of photosynthesis [52]. Over such periods, corals may be forced to consume previously deposited biomass to facilitate genet survival [53,54]. Survival in such environments may therefore favour organisms with larger biomass per unit surface area [55–57], a property that in turn may be facilitated by reductions in coral expansion and/or in rates of secondary calcification [57–59].

By contrast, on the reef slope of Heron Island, temperature and pCO_2_ conditions are thought to be relatively stable over daily time scales due its proximity and exchange with external ocean waters [60]. Light, however, attenuates significantly with depth and can constrain photoautotrophy. On the other hand, the increased exposure to high energy of waves tends to limit the potential for net colony expansion, especially for corals that deposit less dense skeletons over the long term [61,62]. The combination of fragmentation, driven by high wave energy and rapid rates of skeletogenesis can spread the risk of genotype mortality across a larger area [3]. Therefore, it may be hypothesized that, in general, fast growing morphotypes are more frequently associated with high energy locations; whilst fleshier morphotypes, that tend to invest more in tissue than skeletal expansion, are associated with more marginal, low energy habitats [63].

We ran a reciprocal field experiment on *A*. *formosa* collected from in two distinct habitats. We aimed to test an alternative hypothesis that corals survive highly variable environments because they minimise extension rates that enable them to invest their energy into tissue maintenance and production (change in protein density for fixed extension rates). We further aimed to explore whether there was any influence of prior environmental history (i.e. potentially epigenetic memory) that might constrain the responses of colonies of *A*. *formosa*. Specifically, we sought to: (i) determine whether exposure to higher abiotic variability in prior life history increases coral survival irrespective of the characteristic of the new environment; (ii) Whether properties that potentially increase survival are traded for reduced rates of calcification (extension or densification rates); (iii) whether light reductions associated with alteration in depth and reef location, led to a rapid acclimation response (ability to add greater quantities of both protein and calcium carbonate) or not and whether a potential decrease in energy acquisition occurs as corals face changed environmental conditions.

## Materials and methods

### Study site

Heron Reef (23°27’S, 151°55’E) is a lagoonal platform reef which is part of the Capricorn Bunker group located on the southern end of the Great Barrier Reef, Australia. Past sea surface temperatures (SST) for the sea surrounding Heron Reef had a maximum monthly mean (MMM) of 27.3°C (between 1985 and 2001; Weeks, Anthony [64]). This is also the MMM used to determine hotspots and degree heating weeks (DHW), and thereby exposure to thermal stress [65]. For this study, one site was located on the reef flat, and one site on the outer reef slope. Both are on the southern side of Heron Island. Conditions on the reef flat [1-3m, 66] are highly variable with regular periods of ponding with minimal or no inward or outward exchange of water [15,67–70]. Heron Reef has a semi-diurnal tide cycle, which drives a large scale variation in temperature, oxygen, and carbon dioxide [17]. The reef slope site (depth 7-8m at mid-tide, Harry’s Bommie, dive site Heron Island) is on the outer margin of the reef nearby (~0.5km) Wistari channel and hence exposed to well mixed ocean waters along with significant wind and wave activity [71,72]. Brown, Bender-Champ [16] conducted studies at the same locations finding that mean photosynthetically active radiation (PAR) was two to three times higher on the reef flat than the reef slope throughout the year, and more variable (Reef slope mean = 69.35 μmol quanta m^-2^s^-1^, SD = 265.24, Reef flat mean = 394.74 μmol quanta m^-2^s^-1^, SD = 564.92). Mean temperature at the reef flat and slope locations remained similar between sites seasonally but was significantly more variable on the reef flat (Reef slope mean = 24.32°C, SD = 0.84, Reef flat mean = 24.32°C, SD = 1.15). Heron Reef is dominated by *Acropora* spp. [16,51] which are typically fast growing under normal conditions, and as a result can rapidly recolonise after events that have led to reef degradation [3], especially via fragmentation, making them fundamental in maintaining positive carbonate balances within coral reef communities [73].

### Reciprocal transplantation

**Fig 1:**
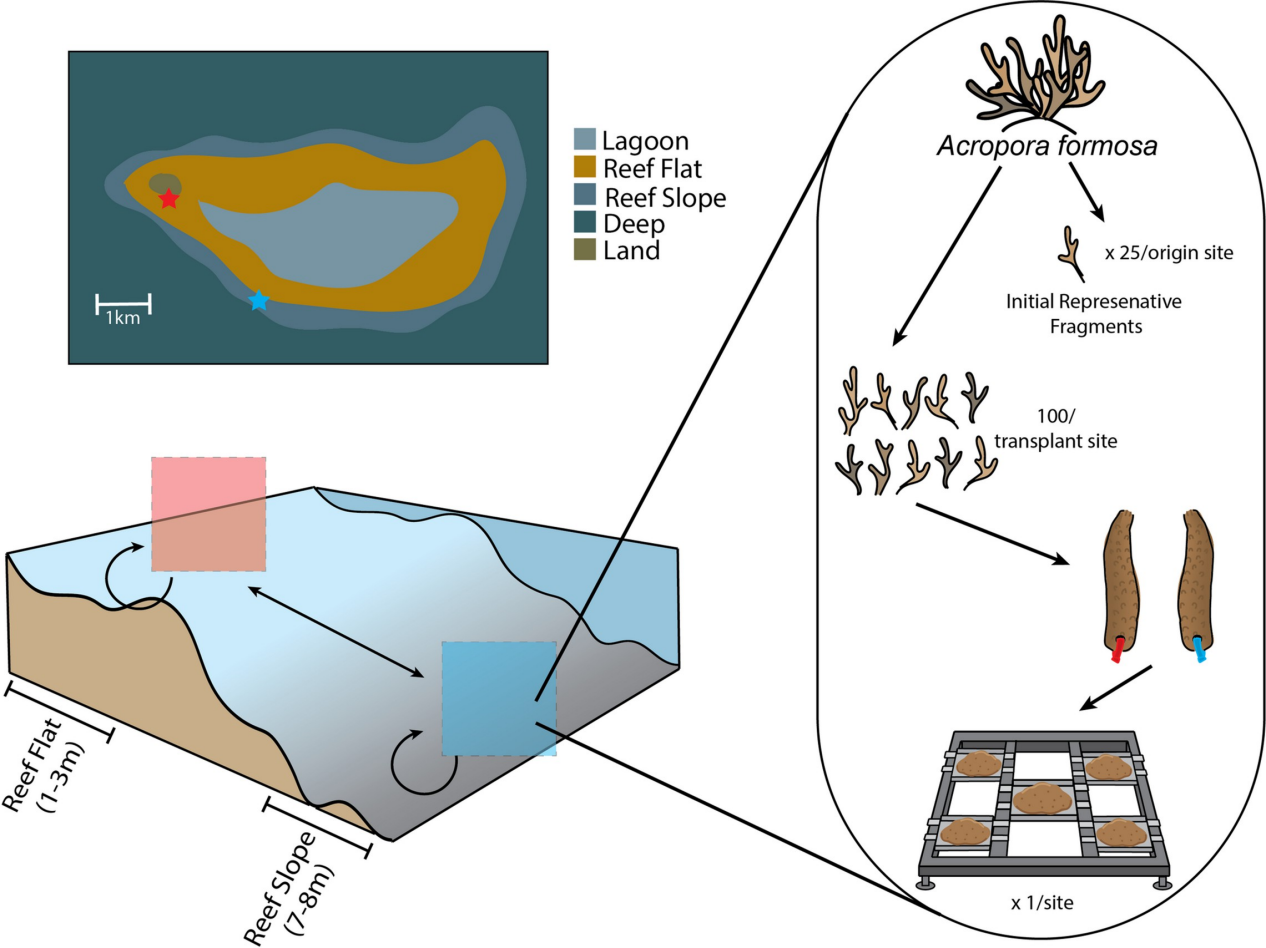
Coral fragments were transplanted between locations on the reef flat (red) and reef slope (blue) of Heron Island in the Southern Great Barrier Reef of Australia. For clarity, sites of colony origin are referred to as “reef slope” (blue zip tie) and “reef flat” (red zip tie), and locations of experimental positioning (transplantation) are referred to as “deep” and “shallow”, however, they describe the same habitat. In total 250 fragments of *Acropora formosa* were collected, 25 of these were stored as representative fragments to be used for initial measurements. The remaining 200 fragments had holes drilled and zip ties threaded through; these were attached to live rock for transplantation onto a metal rack. Created in Adobe Illustrator.

Fragments (7-10cm) of *A*. *formosa* were collected from different colonies, roughly 5m apart, at each location (Flat n = 125; Slope n = 125) in late September 2017. Fragments were randomly collected from the growing tips of colonies fragments with axial branches or damaged apical corallites were excluded. While these were selected from different colonies to avoid pseudo-replication the colony from which a fragment was collected was not tracked. Corals were kept in flow through aquaria under a shade cloth for 2 weeks during initial measurements and preparation for the different experimental treatments. All fragments were trimmed to 7cm using bone cutter scissors. A total of 25 fragments from each location (Flat, Slope) were sampled to assess initial coral condition. These fragments were stored at -20°C and analysed at Heron Island Research Station and/or the University of Queensland. A hole was drilled ~1cm from the base of each fragment intended for reciprocal transplantation, in order to attach the coral to pieces of flat live rock (~25cm x 25cm) for outplanting, 20 corals were attached to each rock. Live rock was used to stabilise fragments rather than plastic or metal structures to limit algal growth and potential competition (Fig 1). Fragments were randomly assigned to treatments in a fully crossed design (Fig 1), corals originating from the reef flat were either returned to the reef flat (“Origin[Flat]/ Transplant[Shallow]”, n = 50), or transplanted to the reef slope (“Origin[Flat]/ Transplant[Deep]”, n = 50), and corals originating from the reef slope were either returned to the reef slope (“Origin[Slope]/ Transplant[Deep]”, n = 50), or transplanted to the reef flat (“Origin[Slope]/ Transplant[Shallow]”, n = 50). Once attached to live rock corals were deployed on metal frames at each location in late September 2017 (1 frame per location, n = 2). The live rocks with fragments attached were collected in late November 2017 for observation. All dead corals were removed (n = 24) prior to redeployment. Final collection of the fragments was conducted in mid-February 2018, each fragment had final buoyant weight measurements taken before being stored at -20°C until further analysis could be undertaken. Specific methodologies for individual measurements are described below. Buoyant weight changes are reported as total change over time of each individual fragment. All other physiological measurements are reported as total change over the experimental period based on the average value calculated from the 25 fragments collected and processed at the beginning of the experiment.

### Physiological analyses

Calcification rates were calculated from initial and final buoyant weight measurements of each individual fragment. Buoyant weight (BW) was measured using methods described by Spencer Davies [74], and Jokiel, Rodgers [75]. BWs were not converted to dry-weight equivalents as this requires an assumed coral density [76]. Changes in wet BW can be driven by calcification contributions to extension (increase in volume or surface area) or densification (BW changes for fixed extension rate). Coral tissue was removed from the skeleton by airbrush using 0.06 M phosphate buffer solution (PBS). Skeletons were then cleaned using a 10% bleach solution to remove any residual tissue. If corals had any tissue die-back this was marked on skeletons in preparation for living surface area analysis. Fragments were rinsed and then dried at 70°C for two hours. The modified double wax dipping method described by Holmes [77] was used to measure the surface area of living tissue. Paraffin wax was heated to 65°C, and coral fragments were maintained at room temperature throughout. This method has been shown to give the most accurate surface area estimation compared to X-ray CT scanners for Acroporid corals [78,79]. Methods for determining bulk volume were based off calculations described in Bucher, Harriott [80].

$$V_{enclosed}=\left(DW_{wax}-BW_{wax}\right)*\partial_{m}$$ Eq 1

where V = volume, *DW*_*wax*_ = dry weight of skeleton with single coat of wax, *BW*_*wax*_ = buoyant weight of skeleton with single coat of wax, *∂*_*m*_ = density of the fluid medium.

The coral tissue in PBS was centrifuged at 4500rpm for 5 minutes (3K15 Sigma laborzentrifugen GmbH, Osterode, Germany), to separate the symbiont pellet. The resulting supernatant was stored in two parts to allow for protein and lipid analyses. For protein analyses the supernatant was analysed with a spectrophotometer (Spectramax M2 Molecular Devices, California, USA), using absorbance values at 235nm and 280nm. Protein densities were then calculated using equations in Whitaker and Granum [81]. For lipid analyses, the supernatant was stored at -80°C overnight. Frozen samples were placed in a freeze dryer (Coolsafe 9l Freeze Dryer Labogene, Allerød, Denmark) at -110°C for 24 to 48 hours until all moisture was removed. Total lipid extraction from each sample was conducted using methods described in Dunn, Thomas [82].

### Mortality

Fragments in the reciprocal transplant could only be identified as dead in collection periods (2.5 months after the start of the experiment, and the end of the experimental period), at which point they were removed from the live rock. At the midway point of the study, 24 corals were classified as dead (defined as 100% tissue loss) and removed, with one additional coral removed due to mortality at the end point of the study.

**Fig 2:**
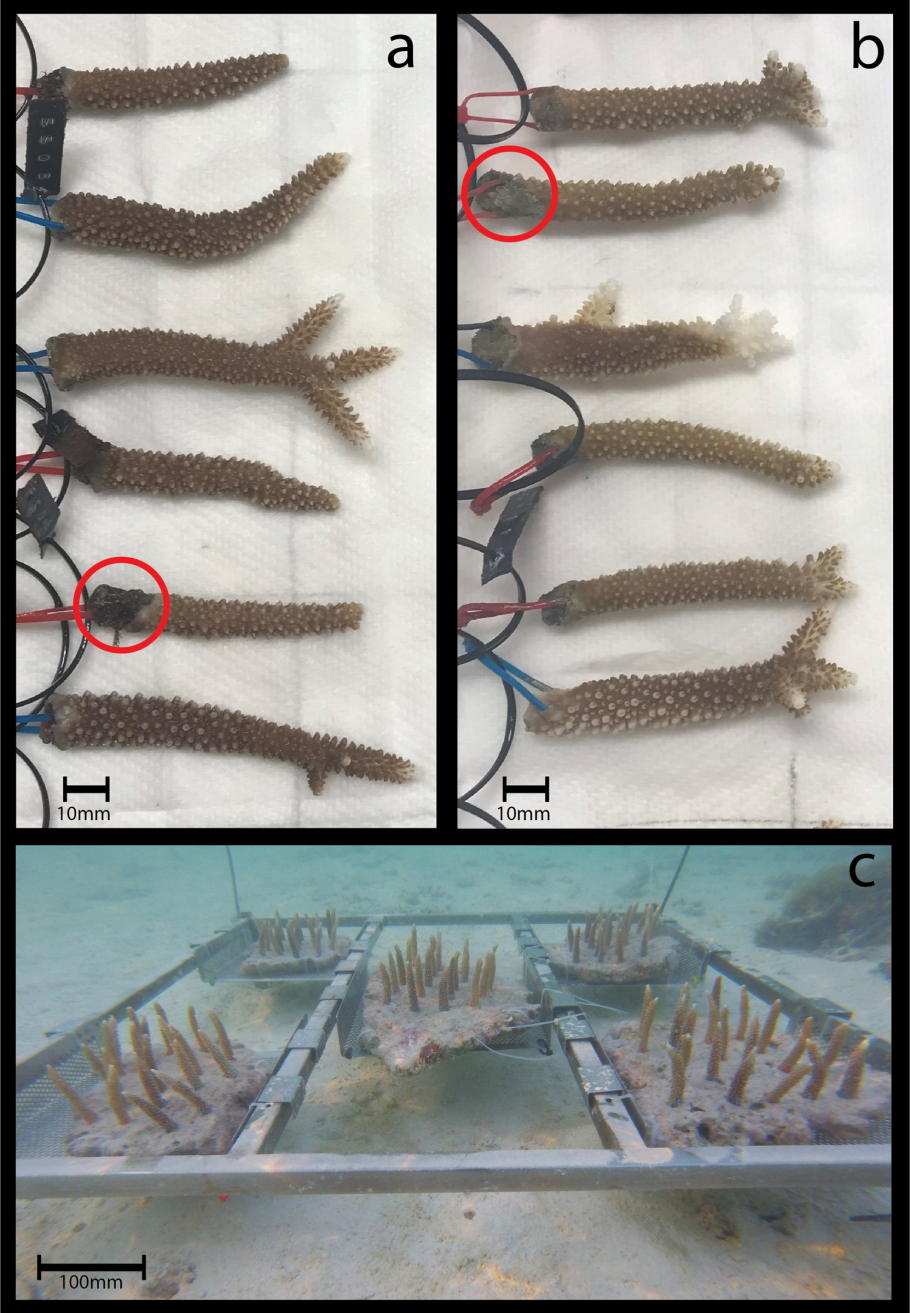
End-point examples of *Acropora formosa* fragments following a reciprocal transplantation experiment conducted at Heron Island (Southern Great Barrier Reef). (a) Fragments experimentally located as transplants to the “deep” reef slope, and (b) Fragments experimentally located as transplants to the “shallow” reef flat. Zip-ties represent location of colony Origin (slope = blue, flat = red). Tissue dieback can be observed at the base of fragments where zip-ties have been attached, differential levels of dieback are due to a lack of uniformity in the live rock corals were attached to. The red circle in (a) highlights dieback with algal growth, and in (b) highlights dieback with the absence of coralites and tissue. (c) in situ photograph of experimental rack on the reef flat.

Minor tissue dieback (less than 5%) on all live fragments was also assessed at the end of the experiment. Approximately 80% of shallow transplants, and 77% of deep transplants experienced a small (<1cm, shown in Fig 2A and 2B) amount of dieback near the point of attachment to the live rock. Algal growth was also apparent in some cases (Fig 2A), and the absence of coralites and tissue in others (Fig 2B). This was marked and accounted for when conducting live surface area measurements. Additionally, 5% of corals transplanted to the deep exhibited tissue dieback beyond the attachment region but not total mortality, these fragments were excluded from the analysis of physiological properties as accurate measurements could not be taken.

### Environmental variables

Field environmental variables were measured using temperature (Hobo pendent logger Onset, MA, USA), and photosynthetically active radiation (PAR; Odyssey logger Dataflow systems LTD, Christchurch, NZ) loggers. Three PAR and three temperature loggers were placed at each location (“deep” reef slope, and “shallow” reef flat) each within 5 metres of the metal frames on which the corals were attached. On the reef slope, two of the three PAR loggers failed and as a result only one set of data was able to be used for analyses. PAR loggers were fitted with a copper washer around the sensor to prevent biofouling and were cleaned at the midway point when corals were collected for measurements. Temperature loggers collected data as hourly means; PAR loggers collected data as integrated values every 2 hours. For analyses, 24h daily means, 25^th^ percentile (Q1) and 75^th^ percentile (Q3) were determined for temperature, 12h (6h – 18h) daytime mean, Q1 and Q3 were determined for PAR.

### Statistical analyses

Host protein was used as a proxy for host biomass, as has been used across multiple peer reviewed studies [83–85]. We used our statistical analyses to distinguish skeletal extension and densification; where either increase in volume or surface area (SA) were proxies for skeletal extension rates. We then modelled, the impact of Habitat and Origin on buoyant weight (BW) changes applying either Volume or SA as a covariate. This allows us to partition BW into extension and densification components. The slope of BW to SA provided an estimate of BW that went into the extension component. BW responses of the other predictors are then estimated for an unchanging extension rate (fixed) and therefore the BW change for these predicators represents a change in densification of the skeleton. Energetic or resource limitations in corals were observed as the necessity to trade between increasing biomass and one or both parameters of calcification. Abiotic conditions that lead to accelerated rates of calcification without compromising biomass were assumed to be energetically positive [58,86,87].

All statistical analyses were conducted with R version 3.6.2 software (2018), figures were created using the ggplot2 package [88] and sjPlot package [89]. All physiological data (bulk volume (BV), living surface area (LSA), buoyant weight (BW), total protein, and total lipids) were initially calculated as total change over time, however after comparison of initial bulk volume between locations of origin, these were converted to percentage change. Linear mixed effects (LME) models were then developed using a stepwise procedure, Akaike information criterion was applied in each case to select the model with the best fit. Each model was assessed for normality and homogeneity of variance through visual inspection of Q-Q plots and residual vs fitted values prior to analysis. Models with different response variables were built up in order of increasing complexity in order to explore trades between variables (Table 1). The function Anova [90] was applied to models, with the type 2 or 3 selection of treatment of sum of squares based on whether optimal models suggested significant interactions amongst variables.

**Table 1:** Summary of statistical models.

Response Variable	Predictors (Initial Model)	Predictors (Best Fit Model)	Statistical Analysis
Mortality	Transplant * Origin	Origin **(F<Sl)**	GLMER
Bulk Volume	Transplant * Origin	Transplant **(Sh>D)** + Origin **(F<Sl)**	LME
Living Surface Area	Transplant * Origin + BV	Transplant **(Sh>D)** + Origin **(F<Sl)** + BV **(+)**	LME
Buoyant Weight	Transplant * Origin + BV/LSA	Transplant * Origin + LSA **(+)**	LME
Total Protein	Transplant * Origin + BV/ LSA + BW	Transplant * Origin + LSA **(+)**	LME
Total Lipids	Transplant * Origin + BV/LSA + BW	Transplant * Origin + LSA **(+)**	LME
Mean PAR	Date * Location	Date * Location	GLS
IQR PAR	Date * Location	Date * Location	GLS
Mean Temperature	Date * Location	Date **(+)** + Location	GLS
IQR Temperature	Date * Location	Date **(+)** * Location **(F>Sl)**	GLS

For physiological analysis, model response variables were: BV, LSA, BW, total protein, and total lipids all of which were continuous. Of the fixed predictors, Origin and Transplant were categorical, while the remaining (BV, LSA, and BW) were continuous. Due to collinearity as measured through variance inflation factor (VIF), covariates BV and LSA were identified as interchangeable in subsequent models. The model with the best fit was chosen as previously described. All physiological analysis and mortality models included live rock as a random effect, which refers to the live rock on which the coral was placed, this remained constant throughout the experimental period and is representative of the coral’s location in space.

The probability of mortality was analysed as a binomial response variable using a mixed effect logistic regression [GLMER, lme4, 91] to take the random effect of live rock into consideration. Binomial response is based on probability theory, from which we can estimate the probability of an outcome. The probability was then converted into relative risk using the “odds_to_rr” function [89]. Laplacian approximation was used to determine the best fit model. The full and best fit models are shown in Table 1.

A generalized least squares (GLS) model was used to compare environmental variables (Temperature and PAR) between each site (Shallow and Deep) over the four-month experimental period. A GLS model was used as data were identified as being temporally autocorrelated. The daily mean, and interquartile range (IQR, between Q1 and Q3 as previously described), as an indication of variability, were both analysed for temperature and PAR, full and best fit models are shown in Table 1, with date and location as categorical predictors. Degree heating weeks (DHW) were calculated using Q3 to measure heat accumulation over the experimental period. Calculations were based on theory from NOAA (Liu et al. 2014) where heating weeks accumulate when temperatures rise above MMM +1.

Response and predictor variables for the initial and best fit models applied to the analysis of samples from the reciprocal transplantation experiment conducted on Heron Island (Southern GBR). Underlined predictors in best fit model indicate a significant effect (P<0.05), the direction of these significant effects is indicated in brackets of the best fit models. Continuous predictors such as “BV” (+) defines a positive correlation to the response variable. For categorical predictors such as “transplant” the relationship between levels of the factor are provided; Transplant, Sh = Shallows, D = Deep; Origin, F = Flat, and Sl = Slope. The direction of interaction effects is not indicated in this table. Variables are abbreviated in model outputs; bulk volume = BV, living surface area = LSA, buoyant weight = BW.

## Results

### Initial measurements, environmental parameters, and mortality potential

**Fig 3:**
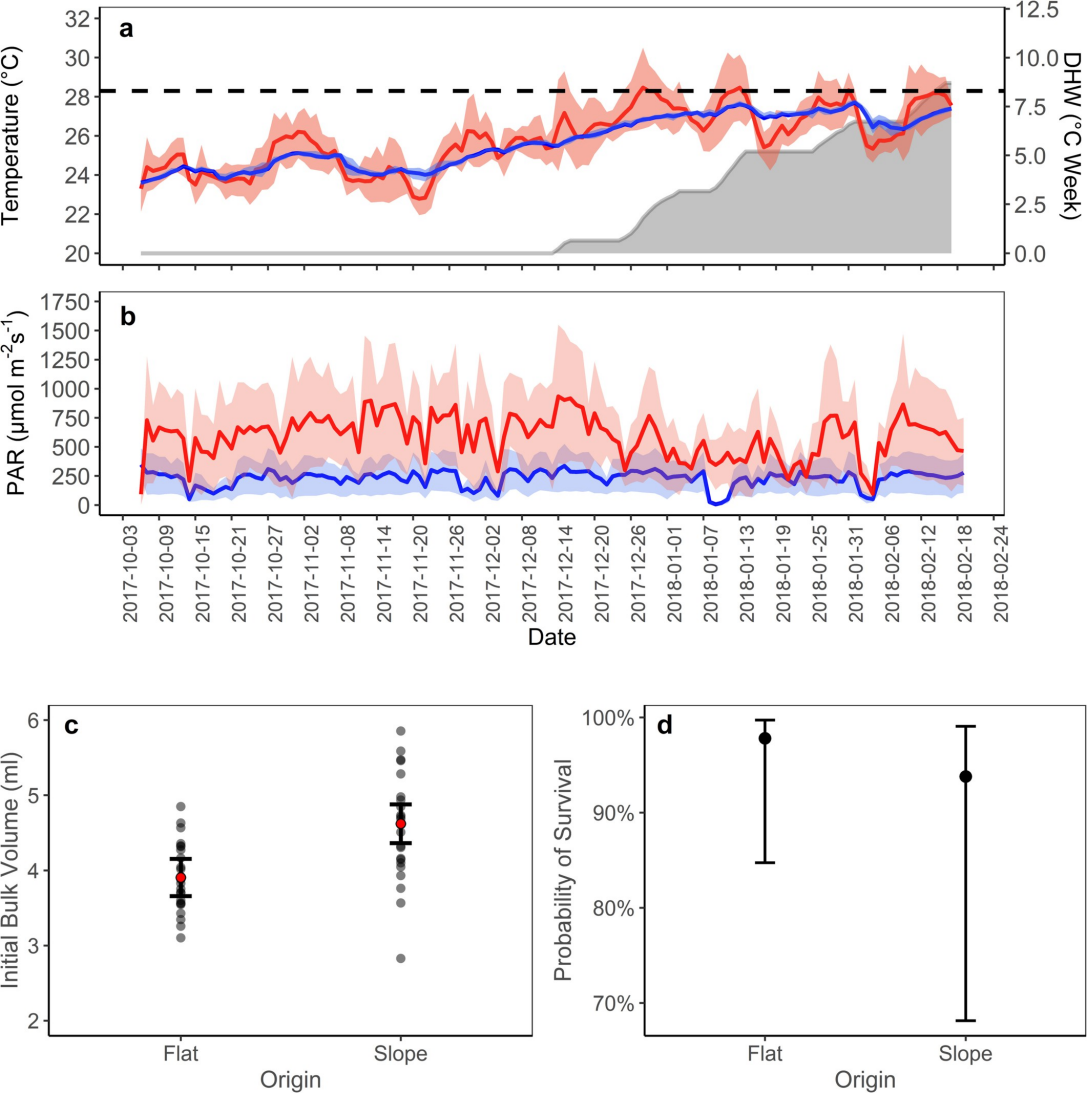
Fragment mortality potential, initial bulk volume of fragments and abiotic conditions associated with the reciprocal transplantation exercise conducted between the reef flat and reef slope of Heron Island (Southern GBR). (a) *In situ* 24hr mean temperature (°C) and (b) *in situ* daytime mean PAR (photosynthetically activate radiation, μmol m^−2^ s^−1^) between 6:00 h and 18:00 h. Abiotic conditions for the deep site (depth = 7-8m, blue line), and the shallow site (depth = 1-3m, red line) were recorded from October 2017 –February 2018. Shading on both abiotic condition figures represent 25^th^ percentile (Q1) on the bottom, and 75^th^ percentile (Q3) on the top, of daily data recorded at each location. The dashed line in figure (a) represents the local MMM + 1 (28.3°C) which is associated with bleaching threshold. Grey area under curve indicates Q3 degree heating week (DHW) accumulation on the reef flat. (c) Initial differences in bulk volume (ml) between *A*. *formosa* fragments from each location of Origin (p<0.05). (d) The probability of fragment mortality by their location of Origin4, error bars represent a 95% confidence interval, grey dots show data points, and red dots indicate the mean value in data set.

Mean temperature increased over time (with the onset of summer) at both sites (Anova, F_(3,290)_ = 830.900, P = *<*0.001, Fig 3A) although mean temperature did not differ between the shallow site and deep site (Anova, F_(3,290)_ = 3.500, P = 0.062, Fig 3A). The variability in temperature (daily interquartile range, IQR), however, was significantly higher in the shallow than in the deep (Anova, F_(3,290)_ = 666.535, P = *<*0.001, Fig 3A). Temperature variability significantly increased at both locations over the experimental period (Anova, F_(3,290)_ = 5.680, P = 0.017, Fig 3A). The Q3, and mean to a lesser degree, periodically breached the MMM+1 (28.3°C) threshold, established for the greater region, in the shallow but not in the deep. The Q3 temperature of the shallow site breached these thresholds for periods that were sustained for less than 2 weeks 4 times over the end of the experimental period. Q3 temperature for the shallow site was associated with heat accumulation maximising as >7.5 degree heating weeks (Fig 3A).

A significant interactive effect was observed between date and location for mean PAR (Anova, F_(3,270)_ = 5.9214, P = 0.0156). Mean daily (06:00–18:00) PAR over the experimental period was always higher in the shallows (Mean = 590.52 μmol quanta m^-2^s^-1^, SD = 178.79) than the deep (Mean = 229.67 μmol quanta m^-2^s^-1^, SD = 68.00). However, amplitude changes in mean daily PAR over successive days were often greater in the shallows than in the deep (Fig 3B). Likewise, variability in PAR, measured as the daily IQR also involved a similar significant interaction between date and location (Anova, F_(3,270)_ = 5.807, P = 0.0166, Fig 3B).

*Acropora formosa* fragments from the reef slope had significantly greater initial bulk volume than reef flat origin fragments (Anova, F_(1,46)_ = 15.420, P = *<*0.001, Fig 3C). Initial protein density and lipid concentrations did not differ among branches from the distinct habitats (t-test, p <0.300, <0.600, respectively).

Over the length of the experimental period, corals that originated from the reef slope, irrespective of their transplant location, were 20% less likely to survive than reef flat origin corals (Anova, *χ*^2^ = 4.604, d.f. = 1, *P* = 0.032, Fig 3D). This occurred irrespective of the random effect of live rock (fragment position in space) on mortality. Over the course of the 5-month experimental period 25 coral fragments perished. All were transplanted to the deep. Of these 25 fragments, 8 originated from the reef flat, and the remaining 17 from the reef slope.

### Physiological response

**Fig 4:**
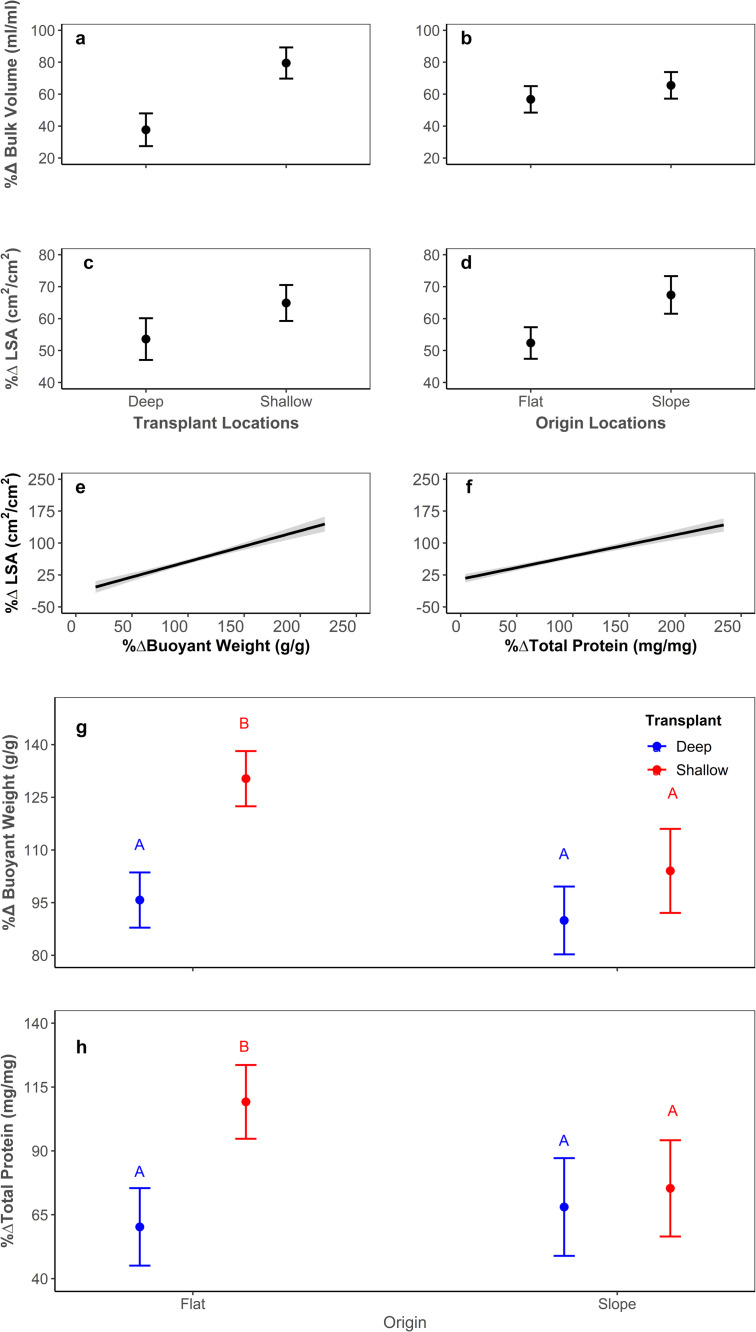
Change in bulk volume (ml/ml) of *A*. *formosa* fragments form Heron Island. (Southern GBR) by (a) location of Transplantation (P <0.05), and (b) location of Origin (P <0.05). Change in living surface area (LSA, cm^2^/cm^2^) of *A*. *formosa* fragments by (c) location of Transplantation (P<0.05), and (d) location of Origin (P<0.05) for fixed rate of change in volume. The percentage change in buoyant weight for (e) percentage change in living surface area and (f) percentage change in total protein. For both (e) and (f) the black line indicates mean value, and the shaded band the confidence interval. (g) Change in buoyant weight (g/g) for fixed changes in living surface area between location of Transplantation (shallow and deep), and location of Origin (flat and slope). (h) Change in total proteins (mg/mg) for fixed changes in living surface area between location of Transplantation (shallow and deep), and location of Origin (flat and slope). Error margins in all graphs represent a 95% confidence interval. All variables are reported as percentage relative change from initial over the experimental period, as indicated by “% Δ” in all axis titles.

*Acropora formosa* fragments that were transplanted to the shallow transplant site had a greater percentage increase in relative bulk volume than those transplanted to the deep (Shallow: 79% vs. Deep: 37%, equivalent absolute volume increases of 3.9 mL vs. 2.1 mL; Anova, *χ*^2^ = 33.913, d.f. = 1, *P* = *<*0.001, Fig 4A), irrespective of their location of origin. Location of origin had a separate significant effect on percentage change in relative bulk volume of fragments, those originating from the reef slope increased their bulk volume significantly more than those from the reef flat (Slope: 65% vs. Flat: 56%, or 4.1mL vs. 2.3 mL; Anova, *χ*^2^ = 4.026, d.f. = 1, *P* = 0.045, Fig 4B).

Analysis of relative rates of change in living surface area (LSA) found that when other parameters were held constant at mean value: (i) rates of change in LSA correlated positively with rates of change in coral volume (Anova, *χ*^2^ = 171.814, d.f. = 1, *P* = *<*0.001); (ii) rates of change in LSA were significantly greater in corals transplanted to reef flat compared to those transplanted to reef slope (Shallow: 64% vs. Deep: 53%, equivalent absolute surface area increases of 19.2 mm^2^ vs. 7.1 mm^2^; Anova, *χ*^2^ = 5.601, d.f. = 1, *P* = 0.018, Fig 4C); (iii) rates of change in LSA were significantly greater in corals of reef-slope origin than in corals of reef-flat origin (Slope: 67% vs. Flat: 52%, equivalent absolute surface area increases of 18.01 mm^2^ vs. 10.0 mm^2^; Anova, *χ*^2^ = 14.708, d.f. = 1, *P* = *<*0.001, Fig 4D). For the same rate of change in volume, changes in coral LSA were greater in reef slope origin corals and tended to indicate increased surface rugosity as opposed to reduced partial tissue mortality.

Relative rates of change in buoyant weight (BW) between *Acropora formosa* fragments analysed when other parameters were held constant at mean value found that: (i) rates of change in BW correlated positively with rates of change in fragment LSA (Anova, *χ*^2^ = 24.383, d.f. = 1, *P* = *<*0.001, Slope = 0.45 +/- 0.06%BW/%SA; (ii) corals that originated from the reef flat and were transplanted to the shallow (“Flat/Shallow”; 130%, equivalent absolute BW increases of 3.5g) had significantly higher rates of change than any other group (Anova, *χ*^2^ = 4.980, d.f. = 1, *P* = 0.026, Fig 4E), all other treatments (“Flat/Deep”, “Slope/Deep”, and “Slope/Shallow”; 95% vs. 89% vs. 104%, equivalent absolute BW increases of 1.6g vs. 2.4g vs 2.9g) were not significantly different from each other.

Analysis of relative rates of change in total proteins found that when other parameters were held constant at mean value: (i) rates of change in total proteins correlated positively with rates of change in LSA (Anova, *χ*^2^ = 6.342, d.f. = 1, *P* = 0.012); (ii) rates of change in total proteins were significantly greater in fragments originating from the reef flat transplanted to the shallow, than transplanted to the deep (“Flat/Shallow” 102% vs “Flat/Deep” 64% equivalent absolute protein increases of 50.8mg vs 9.4mg; Anova, *χ*^2^ = 5.046, d.f. = 1, *P* = 0.025, Fig 4F), but not different between reef slope origin corals transplanted to the shallow or deep (“Slope/Shallow” 74% vs “Slope/Deep” 72% equivalent absolute protein increases of 44.7mg vs 28.8mg).

Relative rates of change in total lipids of *A*. *formosa* fragments when analysed with other parameters held constant at mean value were significantly correlated with LSA (Anova *χ*^2^ = 11.948, d.f. = 1, *P* = *<*0.001). But showed no significant effect of fragment origin or transplant location.

## Discussion

We hypothesized that long-term exposure to the highly variable reef-flat environment would both increase survival potential and engender a conservative growth response, where greater biomass is accumulated at the cost of conserving primary calcification rates. A conservative response that would be maintained upon transplantation to the new reef slope environment. The results of our study supported our hypotheses, and thereby, suggest that any potential epigenetic memory implanted by exposure to long-term prior stress is unlikely to be a panacea that will save carbonate coral reefs in the short-term. It is highly likely that reef-flat coral are better survivors irrespective of habitat because they have reduced rates of primary extension. In the present study, reef-flat native coral properties were maintained over 5 months but removal to the less stressful reef-slope environment did not coincide with an increase in rates of calcification. These findings are consistent with previous studies using *Porites cylindrica* at the same locations which maintained native properties over 21 months [36]. Other studies have demonstrated that epigenetic memories can remain in some organisms for generations [12] however, there is currently limited evidence for this in corals [92]. Consequently, the reductions in the rate of calcification have the potential to reduce carbonate coral reef resilience, the ability of carbonate reefs to bounce back from damage incurred from any disturbance events that engenders a loss of 3-dimentional framework or carbonate from the reef ecosystem [93]. The resilience of shallow tropical reefs may slide towards that of deep-cold water reefs that are able to establish as carbonate reefs, over tens of thousands of years, only because disturbance events are rare (presently, mostly man-made and occur in the form of drag-nets), and reef erosion rates scale with reef calcification rates [5,94,95]. In addition, evidence supports that ocean acidification will reduce rates of calcification by reducing bulk density resulting in negative consequences for coral reefs and their ability to keep up with sea level rise [22].

The environmental contrast between the reef slope and reef flat is exhibited by the difference between the mean and variability of the recorded PAR and temperature. The high variability in temperature on the reef flat observed in this study is comparable to temperature fluctuations observed in studies identifying specific coral populations with superior thermal tolerance [30,96]. The pre-conditioning response is based on the assumption that corals from variable habitats are exposed to high temperatures at the top of their daily variable range each day. It is this exposure that is hypothesized to build bleaching and mortality tolerance to future warming. However, this appears to be a species-specific mechanisms, and in some cases has been observed to produce corals that are more vulnerable to heat stress [97]. To quantify this effect, we monitored the heat stress associated with the upper quartile (Q3) of daily temperatures. We found that Q3-DHWs accumulated to 8°C weeks over the height of summer, but only on the reef flat, not on the reef slope. Despite this apparent difference in heat stress, there was no apparent (visible) bleaching irrespective of coral origins (Fig 2A and 2B), only significant mortality differences by coral origin, but not experimental location. Significantly, the Q3-DHW accumulating over the 5 months of our experiment are notably greater than estimates of a Q3-DHW <3 for the 5 experimental days in Oliver and Palumbi (30) that led to greater bleaching and mortality in conspecific from less variable environments. The key difference being that our experiment allows gradual acclimation to warming rather than abrupt introduction to thermal stress. Current events in the field tend to accumulate heat gradually over prolonged periods [7].

Whilst mean temperature between the reef flat and reef slope did not differ significantly, mean PAR did. Based on these data, we conclude that the primary difference between the deeper slope and shallower flat zones is light. However, there could be additional confounding factors such as differences in the concentration of available dissolved or particulate organic matter that were not measured but have been described in other studies [98–100]. The higher light environment of the reef flat appeared to promote primary calcification, whilst experimental positioning on the deeper slope reduced all aspects of coral growth from primary and secondary calcification to protein accumulation. Nonetheless, reef slope origin corals experienced significantly greater primary calcification than reef flat origin corals at both experimental locations. Greater light and/or greater availability of organic nutriment may increase the energy available for growth processes to reef flat located corals relative to reef slope located corals over the long-term.

Over the experimental period of 5 months, reef flat origin corals showed a reduced ability relative to corals originating from the reef slope to acclimate to their new environment. Reef flat origin corals appeared unable to increase their energy acquisition to maintain the additional energy required to increase skeletal and protein densities on the slope, despite their general tendency to exhibit slower volume and tissue expansion rates [58,86]. This could be, either a result of symbionts that do not acclimate to the new light environment, and/or because organic nutrients are less available at the interface between the ocean and the reef, than within a ponding reef-flat where excreted dissolved organic carbon can be enriched by microbial activity [100,101]. By contrast, reef slope origin corals that expand relatively rapidly irrespective of location, were not apparently photo-inhibited or photo-oxidised [102] by the move to the higher light regime, but actually benefited from an apparent increase in available energy observed as a slight increase in rates of secondary calcification. This is contrary to expectations. The literature tends to argue that zooxanthellate corals can be photo-inhibited, potentially resultant from photooxidation, by increases in light, especially if light increases come in combination with increases in thermal stress [102,103]. By contrast, many corals can acclimate over days to weeks to reduction in light [87,104].

A potential reason the corals benefitted from the higher light environment in our study is that the high light habitat is potentially relatively richer in dissolved and particulate organic food [99,100]. Corals with thicker tissues and reduced rugosities, have been linked to a reduced ability of dinoflagellate endosymbionts to respond to external light fluxes due to the probability that the skeleton plays a lesser role in increasing the path-length of these photons [105]. Here, host pigment and other tissue structures compete with symbionts to absorb photons [106,107]. This could be the optimal response of a coral subjected to highly variable light dosing through time, that otherwise may consume significant energy as they rebuild light harvesting structures and repair D1-protein when light fluxes exceed the capacity of short-term responses such as xanthophyll cycling [108,109]. Such hosts may depend more on heterotrophy. The data from our study does not suggest that primary calcification on the reef flat is limited by the available ‘accommodation space’ of water above colonies [41,110,111]. This experiment was performed on small fragments that were not limited by water depth at either location. Furthermore, reef-slope corals translocated to the reef-flat did not show any limitations in their ability to increase extension rates. Increasing water depth, is likely to reduce light levels unless corals have the capacity to keep up, and/or acclimate their energy acquisition in response to the reduction in light [112]. Reef-flat origin corals in this study did not demonstrate this potential.

We have demonstrated here that conspecific of *A*. *formosa* that showed greater potential to survive a diversity of environments appear to exhibit a reduced ability to acclimate to changes in light availability. Attempting to further protect such corals from bleaching by periodically reducing light fields [43], would therefore impart further restrictions on energy acquisition and calcification. By contrast, the translocation of reef slope communities to the reef flat, did not generate any evidence of bleaching, despite the significant increase in the light field and regular incursions into stressful temperatures. Given that the mortality rate of these corals did not vary by transplant location, the evidence suggests that Acroporids originating from the reef slope acclimate to the changing light regime relatively quickly [21 days; 87,109]. Rapid photo-acclimation however has significant ramifications on the efficacy and practicality of applying shade to protect such corals should they be exposed to greater heat stress. Shade is intended to reduce excitation pressure at Photosystem II (PSII) and prevent the photo-oxidation to the Symbiodiniaceae photosystems, but if these photosystems rapidly increase the efficiency of photon capture in response to reductions in the light field, then shade will not serve to reduce excitation pressure. Shading will however consume additional energy as symbionts would need to invest in rebuilding their light harvesting antennae [113,114], potentially reducing quanta of energy translocation to host.

On a more positive note, reef flat origin coral transferred back to the reef flat had higher skeletal density relative to other treatments, whilst also maximising protein density. Consequently, primary calcification was reduced. Increases in skeletal density can reduce fragmentation and over the long-term [115,116], even colonies with limited primary calcification can accumulate to cover a large area in space as long as physical forces do not exceed the strength of the produced skeleton [117]. Notably lower expansion rates would be beneficial to the maintenance of symbiont densities, as the need for production of new symbionts to occupy the new material would be reduced. Rapid outward expansion is reportedly associated with “bleached” coral tissue [118]. Clearly, some of these hypotheses regarding the explanation of the observed responses require testing. If epigenetic memory is responsible for increasing the survival of reef flat corals exposed to highly variable environments, then environments that tandem variability in light and temperature, in addition to many other abiotic variables, result in corals that sacrificed calcification [119,120]. Survival appears not to be based on the reduced ability to bleach, but rather correlates with reductions in volume and tissue expansion in the Acroporid coral tested. Other corals [121], may maintain rates of linear extension, but it is questionable whether other aspects of calcification are not compromised. For example, reef flat and reef slope conspecifics of *A*. *formosa* from this study, clipped to the same length, have different volumes and skeletal densities that forced the use of relative changes to preserve the statistical assumption that covariates are independent of treatment effect.

Presently, we assume that the differences observed between these relatively proximal *A*. *formosa* populations are driven by epigenetic consequences associated with prior long-term exposure to different abiotic environments within the reef-scape. There is, however, the potential that the relative inflexibility of the two coral populations to alter properties when exposed to new environments has a genetic cause in the form of local adaptation. The two populations may hide cryptic speciation, a phenomenon that is increasingly observed amongst coral taxa [33,121,122]. For cryptic speciation to occur amongst spawning populations that are relatively proximal in space, gene flow may be limited [123]. In this regard, temperature can change the timing of spawning [124], and asynchronous spawning can inhibit gene flow [122]. Alternatively, cryptic species could exhibit different larval and settlement properties that reinforce differential recruitment in these zones [125]. In a brooding species, *Seriatopora hystrix* genetic isolation has been observed to result from differences in reproductive timing between populations in deep and shallow habitats [126,127]. More recently the same has been determined for *Pocillopora damicornis* [33]. Interestingly, this genetic differentiation in *P*. *damicornis* was observed on Heron Reef over similar geographic distances as in the current study. However, *P*. *damicornis* is a brooding species while the target species in the current study, *A*. *formosa*, is a broadcasting species.

Corals that have a prior life history of chronic exposure to highly variable environments tend to be less susceptible to mortality. In this study, we observed that corals fragments raised on the reef flat have a greater survival potential in any environment than conspecifics raised on the reef slope. Daily third quartile temperature (3Q-DHW) accumulated to an 8°C week over the summer on the reef flat. In combination with an increased irradiance for reef slope origin corals moved to the reef flat, the new environment however did not increase bleaching susceptibility of these reef slope origin corals. Rather, they demonstrated that coral expansion in these slope origin corals was suppressed by the lower light conditions under which they appear to nonetheless thrive on the reef slope. The greater survival potential of reef flat origin corals was correlated to their having greater tissue thickness (as measured by protein density) likely facilitated by reduced rates of primary and secondary calcification. Furthermore, these reef flat origin corals failed to acclimate to the lower light levels offered on the reef slope as suggested by a lack of energy to maintain high tissue protein densities despite reduced branch expansion. The fact that reef flat origin corals do not physiologically morph into reef slope origin corals upon relocation to the reef slope and vice-versa suggests that these corals have become epigenetically or genetically distinct populations. This study supports the hypothesis that exposure to a highly variable environment results in “tough” corals, but it identifies that one of the costs of increased survival potential is reduced calcification. These corals therefore lack the properties that are required to maintain healthy and functioning carbonate reefs exposed to increases in the intensity and frequency of disturbance events as a result of climate change. Further, they lack the ability to respond positively to the reductions in light field likely associated with future sea-level rise. Branching Acroporids are important to the 3-dimensional structure and many services provided by carbonate reefs [73]. The present study suggests that the characteristic that allow corals to survive and prosper in highly variable reef environments, do not align with those that are required to maintain carbonate coral reefs exposed to the many consequences of climate change.
